# Nephrology Referral and Outcomes in Critically Ill Acute Kidney Injury Patients

**DOI:** 10.1371/journal.pone.0070482

**Published:** 2013-08-02

**Authors:** Verônica Torres Costa e Silva, Fernando Liaño, Alfonso Muriel, Rafael Díez, Isac de Castro, Luis Yu

**Affiliations:** 1 Division of Nephrology, University of Sao Paulo School of Medicine, Sao Paulo, Brazil; 2 Department of Nephrology, Hospital Universitario Ramón y Cajal, Madrid, Spain; 3 Clinical Biostatistics, Hospital Universitario Ramón y Cajal, Madrid, Spain; 4 Instituto Nacional de Investigaciones Agrarias, Madrid, Spain; University of Sao Paulo Medical School, Brazil

## Abstract

**Background:**

Delayed nephrology consultation (NC) seems to be associated with worse prognosis in critically ill acute kidney injury (AKI) patients.

**Design, Setting, Participants, & Measurements:**

The aims of this study were to analyze factors related with timing of NC and its relation with AKI patients' outcome in intensive care units of a tertiary hospital. AKI was defined as an increase ≥50% in baseline serum creatinine (SCr). Early NC and delayed NC were defined as NC performed before and two days after AKI diagnosis day. Multivariable logistic regression and propensity scores (PS) were used to adjust for confounding and selection biases. Hospital mortality and dialysis dependence on hospital discharge were the primary outcomes.

**Results:**

A total of 366 AKI patients were analyzed and NCs were carried out in 53.6% of the patients. Hospital mortality was 67.8% and dialysis required in 31.4% patients (115/366). Delayed NCs (34%) occurred two days after AKI diagnosis day. This group presented higher mortality (OR: 4.04/CI: 1.60–10.17) and increased dialysis dependence (OR: 3.00/CI: 1.43–6.29) on hospital discharge. Four variables were retained in the PS model for delayed NC: diuresis (1000 ml/24 h - OR: 1.92/CI: 1.27–2.90), SCr (OR: 0.49/CI: 0.32–0.75), surgical AKI (OR: 3.67/CI: 1.65–8.15), and mechanical ventilation (OR: 2.82/CI: 1.06–7.44). After correction by PS, delayed NC was still associated with higher mortality (OR: 3.39/CI: 1.24–9.29) and increased dialysis dependence (OR: 3.25/CI: 1.41–7.51). Delayed NC was associated with increased mortality either in dialyzed patients (OR: 1.54/CI: 1.35–1.78) or non-dialyzed patients (OR: 2.89/CI: 1.00–8.35).

**Conclusion:**

Delayed NC was associated with higher mortality and increased dialysis dependence rates in critically ill AKI patients at hospital discharge. Further studies are necessary to ascertain whether this effect is due to delayed nephrology intervention or residual confounding factors.

## Introduction

Acute kidney injury (AKI) occurs with an incidence ranging between 30% and 70% in critically ill patients [1]. Despite several recent advances in AKI assistance, such as refined dialysis support, better dialysis adequacy, volume and fluid management, improved diagnosis and treatment of AKI-related distant organ dysfunction, AKI morbidity and mortality rates remain unacceptably elevated [2]–[9].

An important contribution for the understanding of AKI was the introduction of RIFLE and AKIN criteria [10], [11]. These indices provide a universal AKI definition that allows comparisons among clinical studies worldwide [12]. In addition, the search for earlier and more sensitive renal biomarkers has contributed to a new perspective on early AKI diagnosis and timely interventions [13], [14].

In view of this new AKI clinical approach, early nephrology consultation could provide opportunities for intervention and modification of AKI patients' outcomes. In a pioneer study, Mehta *et al.* reported that delayed nephrology consultation (NC) was associated with increased mortality in critically ill AKI patients but this result was not sustained after adjustment for confounding variables [15]. Recent reports have indicated that early NC may improve hospital-acquired AKI prognosis, although none of these studies have assessed a large group of AKI intensive care unit (ICU) patients [16]–[19]. Additionally, other important aspects, such as determining factors for nephrology referral and optimal time for renal consultation remain to be determined [20].

Therefore, the aims of this study were to analyze the determining factors for nephrology referral and evaluate the hypothesis that timing of nephrology consultation may influence the prognosis of AKI patients in the ICU. We hypothesized that delayed NC could be associated with higher mortality and dialysis dependence at hospital discharge.

## Materials and Methods

Study participants: A prospective, observational study was conducted through an active search for AKI cases by daily visits to six ICUs (pulmonary, internal medicine, trauma, surgical, infectious diseases and emergency) comprising 53 beds at Hospital das Clinicas of the University of São Paulo, a tertiary academic institution in Brazil.

The study protocol was approved by the Hospital das Clinicas of University of Sao Paulo ethics and research committee. An informed consent, either verbal or written, was not required because of the observational nature of the study with critically ill patients in the ICU. The CAPPesq - Comissao de Etica para Analise de Projetos de Pesquisa do Hospital das Clinicas da Faculdade de Medicina da Universidade de Sao Paulo, local ethics and review board waived the need for written informed consent from the participants of the study.

All patients admitted to the selected ICUs were evaluated for AKI during ICU stay, between November 2003 and June 2005. All AKI patients were prospectively followed since AKI diagnosis day until hospital discharge or death. AKI was defined as an increase ≥50% from baseline serum creatinine (SCr), according to the RISK stage of the RIFLE system [10]. Sepsis was defined according to established criteria [21]. Chronic kidney dysfunction (CKD) was considered with an estimated Cr clearance <60 ml/min/1.73 m^2^ (NKF stage ≥III). Surgical AKI was considered as an AKI related to surgical procedures. The exclusion criteria were baseline SCr≥3.0 mg/dL, dialysis treatment, age <18 years old, kidney transplantation, ICU stay shorter than 48 h, AKI diagnosis established more than 24 h before ICU admission, urinary tract obstruction, and hypovolemia responsive to fluids. Nephrology consultation (NC) was classified as early or delayed NC if they occurred before or more than 48 h after the AKI diagnosis day, respectively [15].

In order to guarantee the observational character of the study, data were collected by a single observer, independent of the ICU and nephrology staff, who was not involved in patient care. Nephrology referral was kept by the ICU attending physician discretion. The attending nephrologists run daily rounds and are available 24 h/day. Thus, NC was usually performed immediately after ICU call. Nephrology service is responsible for dialysis support but decisions on dialysis indications are taken together by the ICU and nephrology physicians. The primary outcomes were in-hospital mortality and dialysis dependence at hospital discharge. Hospital and ICU length of stay were also recorded.

The following variables were collected: age, sex, race, hospital and ICU admission days, ICU origin, co-morbidities (assessed by Charlson co-morbidity Index) [22], baseline renal function, presumed AKI etiologies, vital signs, urine output, laboratory tests, vasoactive drug use and mechanical ventilation. Intermittent hemodialysis was defined as dialysis performed for up to 8 hours of duration (Fresenius 4008S, Germany) and continuous renal replacement therapy as continuous venous-venous hemodialysis (CVVHD), prescribed for at least 24 h (Diapact BBraun, Brazil). Illness severity was assessed with the Simplified Acute Physiology Score (SAPS) 3 with the customized equation for the Central and South America (CSA) region [23]. Organ failure was defined as follows: a) respiratory – need of mechanical ventilation; b) central nervous system (CNS) – Glasgow coma scale ≤8; c) hepatic – total bilirubin ≥2.0 mg/dL and/or enzymatic activity (prothrombin time) ≤50%; d) hematological – leukocytes ≤1,000/mm^3^ and/or platelets ≤20,000 and/or hematocrit ≤20%; and e) cardiovascular – cardiovascular SOFA score ≥3 [24].

Statistical analysis: Data were expressed as mean ± SD or median with 25^th^ and 75^th^ interquartile ranges (IQR) as appropriate. Categorical variables were expressed as proportions and were analyzed with Pearson's χ^2^ test for independent groups. Fisher's test was used where appropriate. Multivariable logistic regression models were constructed with backwards variable selection, using *P*-value <0.05 for variable retention. Candidate variables were those with a likelihood ratio of significance <0.05 upon bivariate analysis. The colinearity of the maximal models was evaluated using the criteria proposed by Belsley [25]. Discrimination was assessed using the AUCROC [26]. Calibration was assessed using the Hosmer-Lemeshow (HL) goodness-of-fit test comparing observed vs. expected mortality across deciles of risk [27]. A high *P*-value (>0.05) indicated a good fit of the model.

To address the influence of confounding variables, covariate adjusted propensity scores (PS) were utilized [28]–[30]. A PS for delayed versus early NCs was created with the variables obtained on the AKI diagnosis day. Additionally, a PS for nephrology consultation (yes or no) was created with the variables obtained on the day of the NC or on the third day after AKI diagnosis for those not evaluated by nephrology. A multivariable logistic regression model was generated using factors that differed among groups (*P*<0.05) and a model predicting the likelihood or “propensity” of consultation was fitted. Propensity scores were then incorporated as covariates in a logistic regression model, using mortality as the dependent variable.

A two-tailed *P*-value <0.05 was considered significant. Statistical analysis was carried out using SPSS for Windows, version 18.0 (Chicago, IL, USA), and SAS (SAS Institute, Cary, NC, USA).

## Results

**Figure 1:**
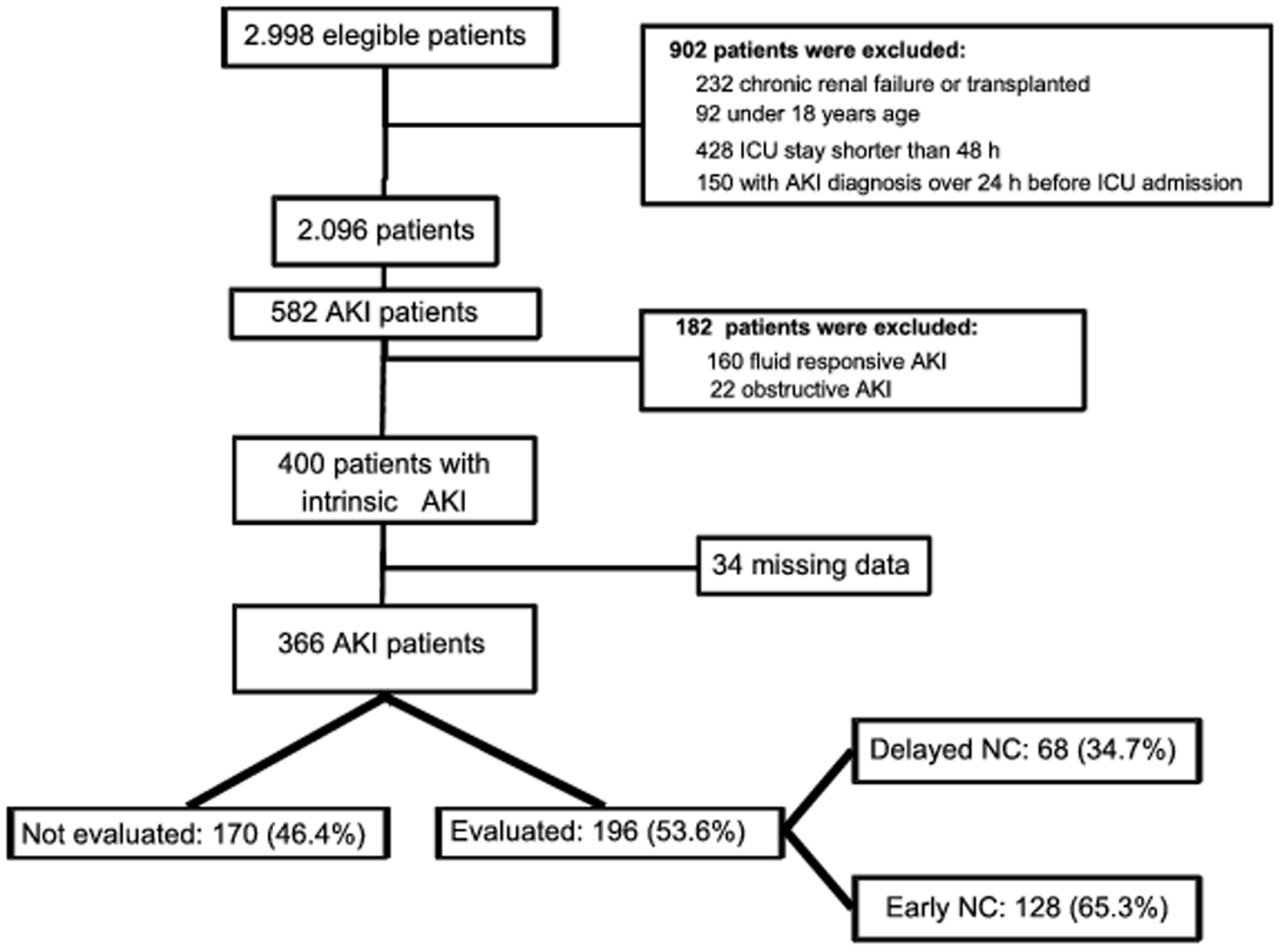
Flowchart of study population. ICU: intensive care unit; AKI: acute kidney injury; NC: nephrology consultation.

During the study period, a total of 2,998 patients were admitted to the selected ICUs. Among these patients, AKI was diagnosed in 400 patients. Only 34 patients (8.5%) were excluded because of missing data, resulting in 366 AKI patients suitable for analysis. NC was performed in 53.6% (196/366) of the AKI patients and occurred 3.0 (1.0–4.0) days after the AKI diagnosis day (DD). Most patients referred to nephrologists were subjected to early NC (65.3%) (Figure 1). The overall hospital mortality was 67.8% (248/366) and dialysis was required in 31.4% (115/366) of patients.

### Nephrology Consultation

Patients referred to nephrologists presented higher mortality (OR: 2.96/CI: 1.87–4.67) and decreased hospital length of stay compared with patients not evaluated by nephrologists (18.5 [11.0–36.0] vs. 27.50 [14.0–37.0] days, P<0.0001). When these two groups (NC and non-NC) were compared on the day of nephrology consultation (day 3), several differences were observed. NC patients demonstrated increased illness severity assessed by organ failures and SAPS3 scores, as well as with parameters related to AKI severity (reduced diuresis and serum bicarbonate [SBic]); increased SCr, blood urea nitrogen and potassium [SK] (Table 1). A propensity score (PS) for NC was created. The five variables retained in the model were: SAPS 3, CSA score, diuresis, SCr, SK, and SBic (Table 2). After correction by the PS, NC was no longer associated with increased mortality (OR: 1.21/CI: 0.66–2.19).

**Table 1:** Patients' characteristics according to nephrology consultation status.

	NC	No NC	
Characteristics *	(n = 196)	(n = 170)	*P* Value
Age (years)	58±18.4	56±19.3	0.320
Male sex	125 (63.8)	93 (54.7)	0.078
Caucasian	135 (68.9)	118 (69.4)	0.912
Surgical AKI	42 (21.4)	44 (25.9)	0.316
Sepsis	137 (69.9)	107 (62.9)	0.159
CKDFCIII	61 (31.1)	32 (18.8)	0.007
Charlson Comorbidity Index^1^	2.0 (1.0–4.0)	2.0 (1.0–3.25)	0.260
Number of organ system failures	3.0 (2.0–4.0)	2.0 (1.0–3.0)	<0.0001
Cardiovascular	105 (53.6)	53 (31.2)	<0.0001
Respiratory	166 (84.7)	117 (68.8)	<0.0001
Hepatic	107 (54.6)	73 (42.9)	0.026
Neurologic	110 (56.1)	74 (43.5)	0.016
Hematologic	24 (12.2)	9 (5.3)	0.038
SAPS 3 CSA^2^	69.7±23.8	53.5±28.5	<0.0001
Serum creatinine (mg/dL)	3.2 (2.3–4.3)	1.7 (1.3–2.3)	<0.0001
Maximum Serum creatinine (mg/dL)	4.30 (3.20–5.30)	2.40 (1.80–3.20)	<0.0001
Blood Urea nitrogen (mg/dL)	55 (38–77)	37(23–51)	<0.0001
Diuresis (ml/24 h)	575 (200–1195)	1645 (937–2662)	<0.0001
Oliguria (diuresis < 400 ml/24 h)	75 (38.3)	17 (10)	<0.0001
Furosemide use (yes/no)	76 (38.8)	35 (20.6)	<0.0001
RIFLE Stage			
Risk	21 (10.7)	40 (23.5)	0.001
Injury	55 (28.1)	63 (37.1)	0.066
Failure	112 (57.1)	37 (21.8)	<0.0001
Serum bicarbonate (mEq/L)	15.0 (13–18)	18 (15–21)	<0.0001
Serum sodium (mEq/L)	141 (136–146)	142 (138–145)	0.314
Serum potassium (mEq/L)	4.9 (4.0–5.5)	4.0 (3.5–4.6)	<0.0001
Total Bilirubin (mg/dL)	0.9 (0.5–2.5)	0.7 (0.4–1.5)	0.055
GOT (UI/dL)	42 (26–99)	36 (21–60)	0.015
Pre-AKI ICU LOS (days)	1.0 (0.0–4.0)	1.0 (0.0–7.0)	0.575
Pre-ICU LOS (days)^3^	4.00 (1.00–14.00)	3.00 (1.00–9.00)	0.268
Dialysis^4^	115 (58.7)	0	<0.0001
Time in mechanical ventilation (days)	12.00 (7.00–19.75)	10.00 (4.00–24.25)	0.149
ICU LOS (days)	16.00 (10.00–25.00)	17.00 (11.00–39.00)	0.066
Hospital LOS (days)	18.50 (11.00–36.00)	27.50 (14.00–37.00)	<0.0001
Mortality	154 (78.6)	90 (55.3)	<0.0001

* Results are expressed in number (%), mean ± SD or median (25–75 IQR).

1 Age points were suppressed.

2 Mortality probability.

3 After hospital admission.

4 Dialysis was exclusively performed by nephrologists.

SD, standard deviation; IQR, interquartile range; CKDFCIII, chronic kidney disease functional class III; SAPS, simplified acute physiology score; CSA, customized equation for countries from Central and South America; GOT, aspartate aminotransferase; LOS, length of stay, AKI, acute kidney injury; ICU, intensive care unit.

**Table 2:** Variables retained in the propensity score model for nephrology consultation (*P*<0.05)*.

	*P*	OR (95% CI)
SAPS 3 CSA^1^	0.002	1.17 (1.06–1.30)
SCr (mg/dL)	<0.0001	2.34 (1.78–3.08)
Diuresis (1000 ml/24 h)	<0.001	0.74 (0.58–0.93)
Serum potassium (mEq/L)	0.037	1.36 (1.01–1.84)
Serum bicarbonate (mEq/L)^2^	0.037	0.53 (0.29–0.96)

* Model performance: area under ROC curve = 0.86 (0.82–0.90); Hosmer-Lemeshow χ^2^ = 0.29.

1 Each 10 units.

2 Each 10 mEq/L.

ROC, receiver operating characteristic; SAPS, simplified acute physiology score; CSA, customized equation for countries from Central and South America; SCr, serum creatinine level; OR, odds ratio; CI, confidence interval.

### Nephrology Consultation Timing – Factors associated with Delayed Nephrology Consultation

AKI diagnosis occurred 1.0 (0.00–4.00) day after ICU admission while NC occurred 3.0 (1.0–4.0) days after the AKI DD. Median time for nephrology referral was 5.5 (4.0–9.0) and 1.0 (0.0–2.0) days in delayed and early NC groups, respectively.

The early and delayed NC groups presented differences, especially regarding AKI origin and renal function on the AKI diagnosis day. There were no differences in demographic (age, co-morbidities) or illness status characteristics (sepsis, organ failure – except mechanical ventilation, severity scoring) between the groups (Table 3). A propensity score for delayed NC was created. The variables retained in the model were diuresis, SCr, surgical AKI and mechanical ventilation (Table 4).

**Table 3:** Patients' characteristics comparing delayed and early nephrology consultation (NC) groups on AKI diagnosis day.

	Delayed NC	Early NC	
Characteristics *	(n = 68)	(n = 128)	*P* Value
Age (years)	60±18	56±18.4	0.20
Male sex	46 (67.6)	79 (61.7)	0.41
White	48 (70.6)	87 (68.0)	0.70
Surgical AKI	23 (33.8)	19 (14.8)	0.02
Sepsis	47 (69.1)	90 (70.3)	0.86
CKDFCIII	22 (32.4)	39 (30.5)	0.78
Charlson Comorbidity Index^1^	5.0 (3.0–6.0)	4.0 (2.0–6.0)	0.301
Number of organ system failures	3.0 (2.0–4.0)	3.0 (2.0–4.0)	0.421
Cardiovascular	59 (86.8)	103 (80.5)	0.128
Respiratory	61 (89.7)	91 (71.1)	0.030
Hepatic	37 (54.4)	70 (54.7)	0.971
Neurologic	36 (52.9)	64 (50)	0.695
Hematologic	5 (7.4)	19 (14.8)	0.023
SAPS 3 CSA^2^	60.7±28.5	60.6±26.0	0.645
Serum creatinine (mg/dL)	1.8 (1.5–2.3)	2.4 (1.8–3.4)	<0.0001
Maximum Serum creatinine (mg/dL)	4.30 (3.31–5.10)	4.20 (3.12–5.30)	0.996
Blood Urea nitrogen (mg/dL)	33 (23–44)	41 (27–63)	0.003
Diuresis (ml/24 h)	1245 (672–1745)	625 (250–1257)	<0.0001
Oliguria (diuresis <400 ml/24 h)	6 (8.8)	48 (37.5)	<0.0001
Furosemide use (yes/no)	17 (25)	37 (28.9)	0.56
RIFLE Stage			
Risk	41 (60.3)	44 (34.4)	<0.0001
Injury	21 (30.9)	39 (30.5)	0.952
Failure	6 (8.8)	45 (35.1)	<0.0001
Serum bicarbonate (mEq/L)	18 (15–21)	15.7 (13–19)	<0.0001
Serum sodium (mEq/L)	140 (136–144)	140 (136–144)	0.74
Serum potassium (mEq/L)	4.6 (3.8–5.2)	4.7 (4.0–5.4)	0.13
Total bilirubin (mg/dL)	0.8 (0.5–2.1)	1.0 (0.5–2.6)	0.28
GPT (UI/dL)	22 (15–50)	31 (17–78)	0.03
Pre-AKI ICU LOS (days)	2.0 (0.5–8.0)	1.0 (0.0–3.0)	<0.001
Pre-ICU LOS (days)^3^	6.5 (1.0–18.5)	4.0 (1.0–11.5)	0.061
Dialysis	34 (50)	81 (63.3)	0.072
Time in mechanical ventilation (days)	16.5 (11.0–32.0)	10.0 (6.00–15.0)	<0.0001
ICU LOS (days)	19.0 (12.5–34.5)	13.5 (9.00–23.0)	0.03
Hospital LOS (days)	20.0 (13.0–36.5)	18.0 (11.0–36.0)	0.493
Dialysis dependence	57 (83.8)	81 (63.3)	0.003
Mortality	62 (91.2)	92 (71.9)	0.002

* Results are expressed in number (%), mean ± SD or median (25–75 IQR).

1 Age points were suppressed.

2 Mortality probability.

3 After hospital admission; SD, standard deviation; IQR, interquartile range; CKDFCIII, chronic kidney disease functional class III; ICU, intensive care unit; CRRT, continuous renal replacement therapy; AKI, acute kidney injury; LOS, length of stay; SAPS, simplified acute physiology score; CSA, customized equation for countries from Central and South America; GPT, alanine aminotransferase.

**Table 4:** Variables retained in the propensity score model for delayed nephrology consultation (*P*<0.05)*.

	*P*	OR (95% CI)
SCr (mg/dL)	0.001	0.51 (0.35–0.76)
Diuresis (1000 ml/24 h)	<0.001	2.24 (1.46–3.43)
Surgical patients	0.001	3.67 (1.65–8.15)
Mechanical ventilation	0.036	2.82 (1.06–744)

* Model performance: area under ROC curve = 0.79 (0.72–0.85); Hosmer-Lemeshow χ^2^ = 0.48.

ROC, receiver operating characteristic; SCr, serum creatinine level; OR, odds ratio; CI, confidence interval.

### Influence of Delayed Nephrology Consultation on patient outcomes

The delayed NC group presented higher mortality (OR: 4.04/CI: 1.60–10.17) and increased dialysis dependence at hospital discharge (OR: 3.00/CI: 1.43–6.29). After correction by the PS, delayed NC was still associated with higher mortality (OR: 3.39/CI: 1.24–9.29) and greater dialysis dependence (OR: 3.25/CI: 1.41–7.51).

Delayed NC patients presented increased ICU length of stay (LOS, days) (19.00 [12.50–34.50] compared with the early NC group (13.5 [9.00–23.00], P = 0.03). Delayed NC patients needed longer mechanical ventilation support (MV, days)) (16.5 [11.0–32.0] compared with the early NC group (10.0 [6.0–15.0], P: 0.001, Table 3). Prolonged time on MV was associated with mortality (8.0 [0.0–18.0] vs. 12.0 [8.0–20.2] for survival and non-survival patients, respectively, P = 0.002).

### Dialysis Characteristics Associated with Delayed Nephrology Consultation

Renal replacement therapy (RRT) was provided exclusively by intermittent hemodialysis (14.7%, 17/115) and CVVHD (87.8%, 104/115). Dialysis was initiated around 4.0 (2.0–7.0) days after AKI DD and 1.0 (0.0–3.0) day after NC. RRT was associated with increased mortality (OR:2.26/CI:1.13–4.53) but RRT requirement was not different in both early and delayed NC groups (P = 0.072).

Time period to start dialysis after NC was not different between early and delayed NC groups (P = 0.054). However, time duration to start dialysis after AKI DD was longer in delayed NC patients (7.0 days [5.0–11.0] than in early NC group (2.0 days [1.0–4.0], P<0.0001). Prolonged time for dialysis initiation after AKI diagnosis was associated with mortality (1.00 [1.0–2.0] vs. 4.0 [2.0–8.0] in survival and non-survival patients, respectively, P = 0.001).

Delayed NC was associated with increased mortality in both dialyzed (31.4%, OR: 1.54/CI: 1.35–1.78) and non-dialyzed patients (OR: 2.89/CI: 1.00–8.35). In addition, delayed NC patients needed longer periods of MV regardless the RRT requirement (Table 5). When dialysis and non-dialysis patients were analyzed on AKI DD, delayed and early NC groups presented similar results: no differences in demographics (age, co-morbidities) or illness status characteristics (sepsis, organ failure, severity scoring). However, early dialyzed NC patients presented worse parameters related to AKI severity such as reduced diuresis and increased SCr (Table 5).

**Table 5:** Patients characteristics comparing delayed and early Nephrology Consultation (NC) subgroups in both dialyzed and no dialyzed patients on Acute Kidney Injury (AKI) diagnosis day.

Characteristics*	Dialyzed patients (n = 115)			No dialyzed patients (n = 81)		
	Delayed NC (n = 34)	Early NC (n = 81)	*P* value	Delayed NC (n = 34)	Early NC (n = 47)	*P* value
Age (years)	58±18.4	57±18.3	0.711	57±17.8	57±18.9	0.807
Male	73 (63.5)	51 (63.0)	0.941	25 (73.5)	26 (55.3)	0.094
Surgical AKI	11 (32.4)	11 (13.6)	0.02	8 (23.5)	10 (21.3)	0.810
Sepsis	82 (71.3)	55 (67.9)	0.609	23 (67.6)	32 (68.1)	0.967
CKDFCIII	39 (33.9)	22 (27.2)	0.315	10 (29.4)	12 (25.5)	0.698
Charlson Comorbidity Index^1^	2.0 (1.0–4.0)	2.0 (1.0–4.0)	0.499	2.0 (1.0–4.0)	2.0 (1.0–4.0)	0.953
Number of organ system failures	3.0 (2.0–4.0)	2.0 (2.0–4.0)	0.098	3.0 (2.0–4.0)	3.0 (2.0–3.0)	0.392
Cardiovascular Failure	104 (90.4)	58 (71.6)	0.001	27 (79.4)	31 (66.0)	0.185
Respiratory Failure	31 (91.2)	59 (72.8)	0.030	30 (88.2)	32 (68.1)	0.035
Hepatic	61 (53.0)	46 (56.8)	0.604	20 (58.8)	26 (55.3)	0.753
Neurologic	64 (55.7)	36 (44.4)	0.122	16 (47.1)	20 (42.6)	0.687
Hematologic Failure	3 (8.8)	12 (14.8)	0.384	2 (5.9)	7 (14.9)	0.203
SAPS 3 CSA^2^	61.2±28.0	59.9±24.7	0.095	60.7±26.0	59.3±23.9	0.892
Serum creatinine (mg/dL)	1.9 (1.5–2.5)	2.5 (1.8–3.6)	<0.0001	1.7 (1.4–2.3)	2.2 (1.7–3.15)	<0.0001
Blood urea nitrogen(mg/dL)	38 (28–54)	48 (31–64)	0.062	28 (21–41)	36 (26–57)	0.069
Serum bicarbonate (mEq/L)	16.9 (14.8–19.6)	14.9 (11.7–17.9)	0.003	16.6 (14.7–18.9)	15.3 (13.7–16.8)	0.113
Serum potassium (mEq/L)	4.7 (3.9–5.5)	4.5 (4.05–5.15)	0.284	4.4 (3.77–5.1)	4.7 (4.2–5.2)	0.283
Diuresis (ml/24 h)	1050 (710–1500)	560 (190–1000)	<0.0001	1345 (660–1920)	800 (340–1700)	0.047
Oliguria (diuresis <400 ml/24 h)	3 (8.8)	34 (42.0)	0.001	3 (8.8)	14 (29.8)	0.022
Time to star dialysis after ICU admission (days)	11.0 (7.0–16.0)	4.00 (2.00–8.00)	<0.0001	-	-	-
Time to start dialysis after AKI diagnosis (days)	7.0 (5.0–11.0)	2.0 (1.0–4.0)	<0.0001	-	-	-
Time to NC after AKI diagnosis (days)	6.00 (4.00–10.00)	1.00 (0.00–2.00)	<0.0001	4.00 (4.00–8.00)	1.00 (0.00–2.00)	<0.0001
Time to start dialysis after NC (days)	1.0 (0.0–2.0)	1.0 (0.0–3.0)	0.053	-	-	-
Pre-AKI ICU LOS (days)	1.0 (0.0–6.0)	0.0 (0.0–3.0)	0.090	3.0 (1.0–11.0)	1.0 (0.0–3.5)	0.002
Pre-ICU LOS (days)^3^	7.0 (1.0–16.0)	3.0 (1.0–8.0)	0.074	5.5 (1.0–19.0)	5.0 (1.0–16.5)	0.516
Time in mechanical ventilation (days)	17.0 (11.0–30.0)	11.0 (7.00–16.0)	<0.0001	15.5 (10.0–35.0)	8.0 (3.50–14.0)	<0.0001
ICU LOS (days)	18.5 (11.0–32.0)	13.0 (9.00–22.0)	0.024	19.5 (14.0–38.0)	16.0 (9.00–23.5)	0.073
Hospital LOS (days)	18.5 (11.0–33.0)	16.0 (10.0–36.0)	0.391	22.5 (14.0–48.0)	22.0 (12.5–34.5)	0.550
Dialysis dependence at hospital discharge	57 (83.8)	81 (63.3)	0.003	-	-	-
Mortality	34 (100)	63 (77.8)	0.003	28 (82.4)	29 (61.7)	0.045

* Results are expressed in number (%), mean ± SD or median (25–75 IQR).

1 Age points were suppressed.

2 Mortality probability.

3 After hospital admission.

CKDFCIII, chronic kidney disease functional class III; ICU, intensive care unit; AKI, acute kidney injury; LOS, length of stay; SAPS, simplified acute physiology score; CSA, customized equation for countries from Central and South America.

## Discussion

In this observational study of critically ill patients, it was observed that around half of AKI patients were referred to nephrologists These patients presented higher mortality which probably resulted from patients' worse illness characteristics (higher SAPS3 score, increased number of organ failures and more severe AKI). However, mortality rate was no longer different between NC and non-NC groups when corrected by the propensity score.

In contrast, a strong association was demonstrated between delayed NC and worse prognosis in NC patients. Mehta *et al.* [15] assessed the impact of NC timing on the prognosis of critically ill AKI patients. Delayed NC was associated with increased mortality in the bivariate analysis but this effect was not sustained after correction by PS. There are similarities compared with our study: ICU setting, similar sample size and PS variables (SCr and diuresis). Conversely, remarkable differences are also present: different AKI definition was utilized because patients were recruited between 1989 and 1995; only patients with AKI diagnosed on ICU admission were enrolled and missing data comprised almost one-third of the initial sample, resulting in only 215 patients out of 822 original AKI NC patients.

Perez-Valdivieso *et al.* [17] published a study with 646 AKI hospital patients using RIFLE definition. They reported that patients who had an increment in SCr greater than 101% at the time of NC presented increased mortality and worse renal function at hospital discharge. However, neither PS nor further statistical analysis was performed, remaining uncertain about the influence of residual confounding factors.

Balasubramanian *et al.* [16] demonstrated that early NC was associated with reduced risk of further decrease in kidney function (RIFLE criteria) in AKI hospital patients, considering a NC timing of 18 hours. For the first time, NC was assessed as an intervention in a randomized trial which probably provided a more powerful statistical analysis. However, informed consent was required for the patients, which might have induced selection bias for the attending physicians and nephrologists. Other limitations of this study were: pre-renal AKI was present in half of the sample; sepsis was the etiology in only one-third of the patients and dialysis requirement was observed in only 1% of the cases. These factors may explain the lack of association of early NC and mortality.

Recently, Ponce *et al.* [18] have published an observational, prospective study assessing 148 AKI ICU patients, in which only 77 patients were evaluated by nephrologists. Delayed NC, using a two-day interval, similarly was associated with increased mortality after adjustment in a multivariable analysis.

In our study, extended time to start RRT after AKI diagnosis was strongly associated with increased mortality. Although this study was not designed to assess decisions about dialysis indications, these data suggest that late RRT initiation may affect patients' prognosis. Decisions about dialysis initiation in the ICU setting are influenced by several factors: time period after AKI diagnosis, AKI severity, AKI etiology, presence of chronic kidney dysfunction and other organ failures, illness severity, volume overload and nutritional status, mechanical ventilation demands, functional reserve (age, co-morbidities) and attending physicians' preferences. An important issue of the present study is that RRT cannot be initiated without nephrology referral. Herein, ICU attending physician's perception about AKI severity and dialysis indication directly influenced nephrology referral and the timing of dialysis initiation.

Patients with delayed NC, regardless of dialysis requirement, presented higher mortality and longer mechanical ventilation support which could indicate either late nephrology intervention or worse patient's clinical status. Although propensity score for delayed NC should have corrected for these biases, we cannot propose that timing itself was responsible for the increased mortality risk.

Propensity score is an important statistical tool but it also has limitations [31]–[34]. Variables included in the model were those statistically significant in the bivariate analysis but other important non-observed variables might have been excluded. Furthermore, this is a single center study and NC was performed by an experienced academic AKI-oriented group with great expertise in AKI management, with all available dialysis techniques that might not be available elsewhere.

The present study has also some strength. An association between delayed NC patients and increased mortality and worse renal function was demonstrated, even after correction for confounding variables in a relatively large group of critically ill AKI patients. Sepsis, the main AKI etiology, was present in almost 70% of patients. Data were collected by a single observer, avoiding inter-observer variability, and only a small group of patients (8.5%) was excluded because of missing data, minimizing selection and analysis biases. Despite of limitations of the propensity score model, it might be a valuable alternative to evaluate the influence of NC on AKI outcome. Alternatively, evaluation of nephrology intervention in AKI patients' outcome in large randomized studies is hard to perform and to reproduce. Indeed, a recent pilot study has not brought better results [16]. Furthermore, our study represents the situation of a routine medical practice in several countries worldwide in which early nephrologists' interventions, including dialysis support, is dependent on ICU physicians' referral to medical specialists and their perception of illness severity and priority.

In conclusion, in this group of critically ill AKI patients, around half of the patients were referred to nephrologists. Despite of the greater severity of NC patients compared to non-NC group, mortality rate was similar when adjusted by the propensity score. In contrast, delayed NC occurred in 34% of AKI patients and it was associated with increased mortality and dialysis dependence at hospital discharge, even after adjustment for confounding factors. A direct causal relationship could not be definitely established because some other factors, such as delayed recognition of severe AKI cases, residual confounding effects and the interface between nephrology and intensive care practice may have influenced these results. Herein, the best medical approach seems to be an early collaborative work between ICU physicians and nephrologists to share decisions and join experiences which could lead to interventions that may influence the critically ill AKI patients' prognosis.
